# Through-and-through nasal infratip defect repair using a salvage rotation flap with standing cone repurposing

**DOI:** 10.1016/j.jdcr.2026.07.050

**Published:** 2026-08-01

**Authors:** Lauren James, Phuong Daniels, Kayd Pulsipher, Chelsea Schwartz, Rene Bermudez

**Affiliations:** a Department of Dermatology, Campbell University/Sampson Regional Medical Center Dermatology Residency Program, Wilmington, North Carolina; b Department of Mohs Micrographic Surgery, Campbell University, Wilmington, North Carolina

**Keywords:** basal cell carcinoma, Burow’s graft, Mohs micrographic surgery, nasal infratip reconstruction, nasal tip rotation flap, secondary reconstruction, through-and-through nasal defect

## Introduction

Reconstruction of through-and-through defects of the nasal infratip following Mohs micrographic surgery presents a unique surgical challenge. The nasal tip has limited tissue laxity and lies adjacent to free margins, making preservation of alar rim support and nasal airway patency critical. Successful repair requires restoration of both the external cutaneous surface and the internal nasal lining while minimizing tension and additional scarring. Common reconstructive options include full-thickness skin grafts, composite grafts, local flaps, and cartilage grafting. However, complications such as graft failure or inadequate structural support may necessitate secondary reconstruction.^1^

Burow’s grafts or rotation flaps are commonly employed in nasal reconstruction, but may be insufficient for full-thickness defects involving the nasal lining. In such cases, salvage reconstruction strategies that utilize local tissue while preserving nasal contour and function are desirable.^2^ We present a case of a persistent through-and-through nasal infratip defect following partial Burow’s graft failure that was successfully repaired using a nasal tip rotation flap with intentional repurposing of the standing cone to recreate the internal nasal lining.

## Case report

A 61-year-old man with Fitzpatrick skin type I presented with a biopsy-proven nodular basal cell carcinoma of the nasal infratip measuring 1.1 × 1.1 cm. The lesion had been present for several months and was asymptomatic. The patient had no history of prior nasal trauma, radiation exposure, or prior cutaneous malignancies. Mohs micrographic surgery was recommended given the tumor location within a high-risk area and poorly defined clinical borders.^3^ Tumor clearance required 3 stages, resulting in a through-and-through final defect measuring 1.5 × 1.3 cm. The nasal mucosa was initially sutured closed with buried 5-0 non-irradiated polyglactin 910 sutures, but the remainder of the reconstruction was delayed due to patient fatigue. The defect was subsequently repaired 3 days later using a Burow’s graft to address the full-thickness nature of the defect.

**Fig 1:**
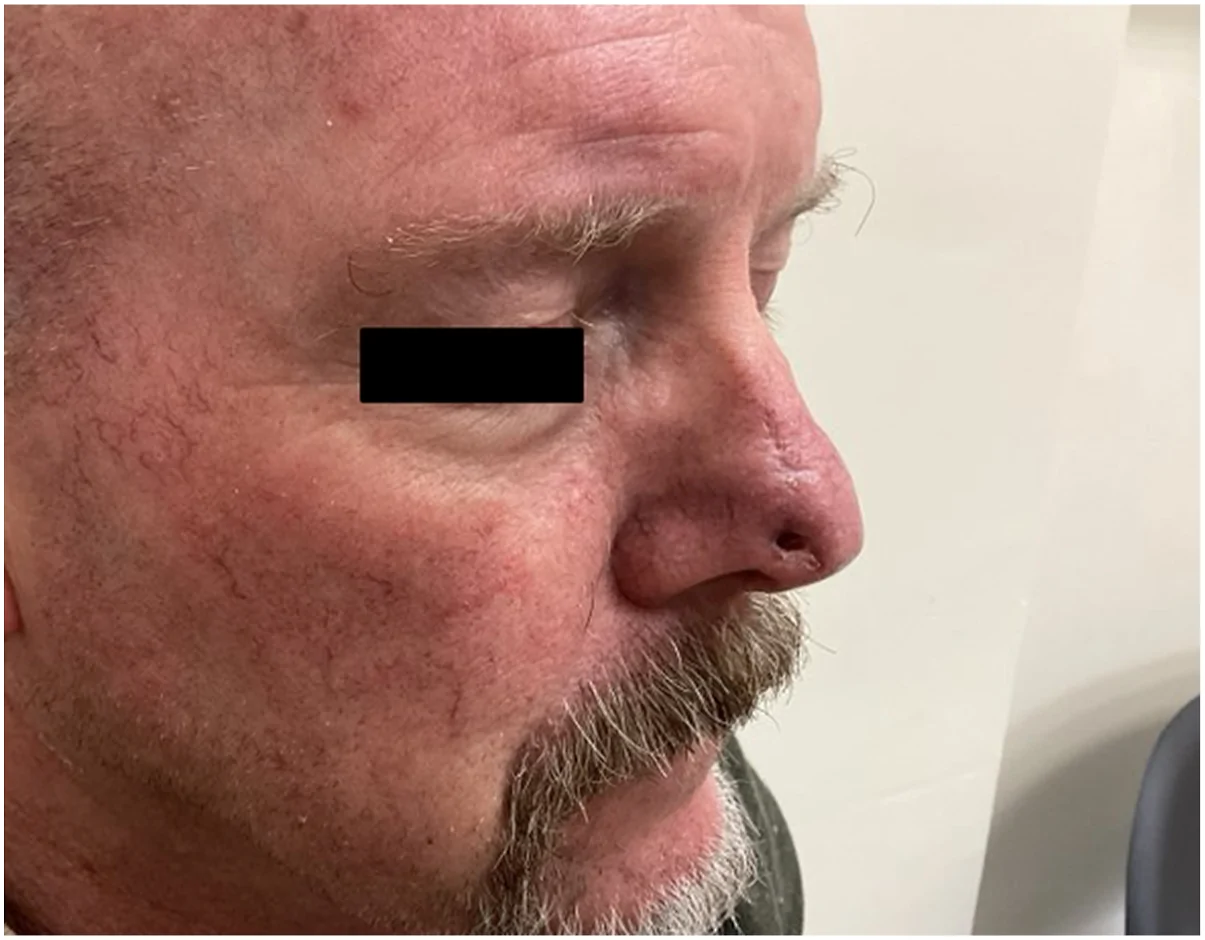
Initial through and through nasal infratip defect.

At the 2-day postoperative follow-up, the patient was doing well with no evidence of infection or graft compromise, and by 14 days postoperatively, healing had progressed appropriately with continued patient reassurance. However, by 52 days postoperatively, it became apparent that the through-and-through defect recurred despite partial graft take (Fig 1). Although the alar rim remained intact, the defect resulted in suboptimal cosmesis, and secondary reconstruction was planned. The goals were to restore the internal nasal lining, preserve alar rim integrity, and minimize further scarring, particularly along the nasal dorsum where the prior surgical scar was present.

**Fig 2:**
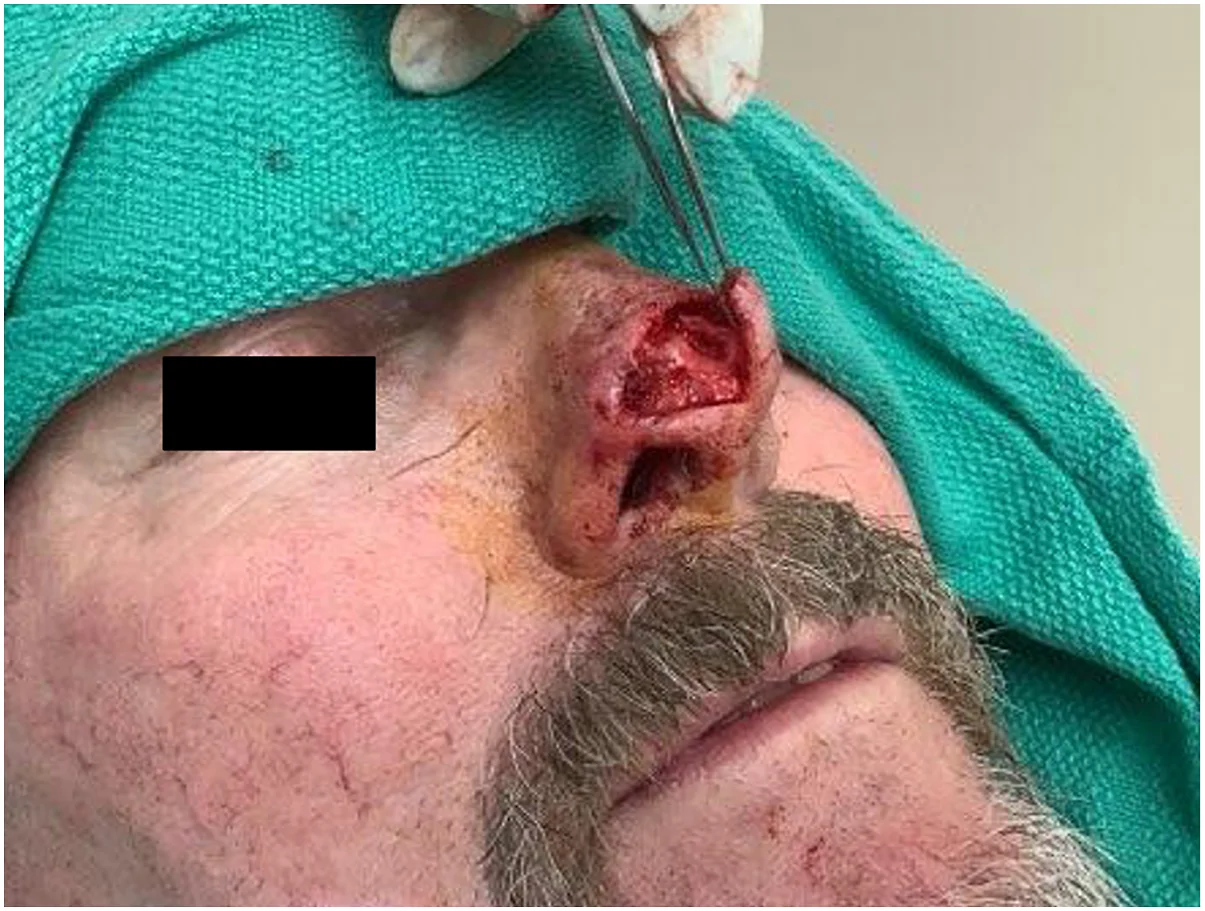
Intraoperative approach utilizing standing cone of tissue from nasal tip.

A nasal tip rotation flap was designed to recruit adjacent tissue while avoiding disruption of the dorsal nasal scar from the initial closure.^4^ The flap was incised to adipose tissue, and the margins were undermined to permit tension-free rotation (Fig 2). The standing cone at the flap apex was preserved and folded inward onto itself in a hinge-like fashion and secured to the base of the defect using buried 5-0 nonirradiated polyglactin 910 sutures. This allowed for reconstruction of the nasal internal lining and provided a base for the overlying rotation flap. This technique avoided the need for cartilage grafting while reducing the risk of alar rim distortion.^5^

**Fig 3:**
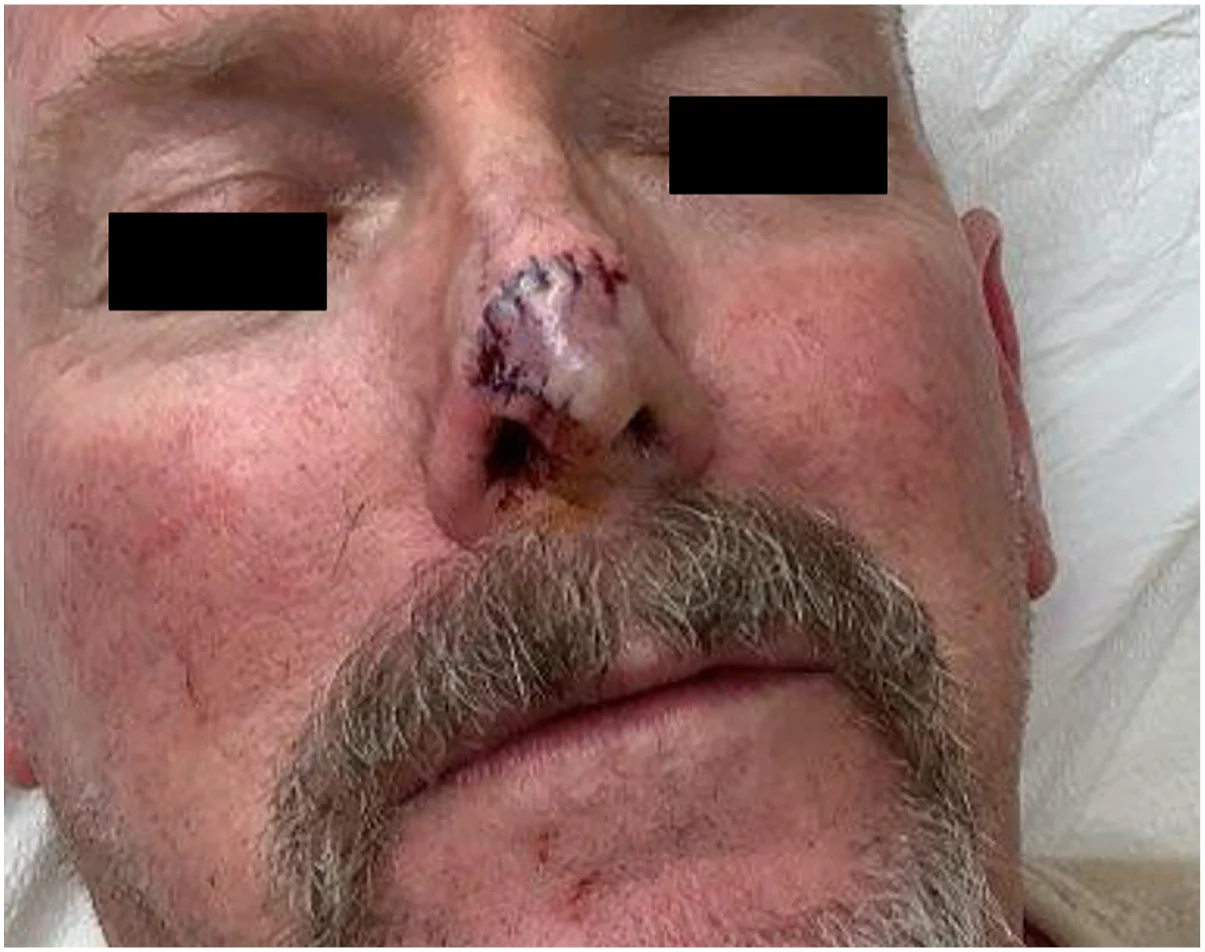
Final closure after utilizing standing cone in rotation flap technique.

Following reconstruction of the internal lining, the flap was rotated over the defect and sutured in place using 5-0 polyglactin for deep tissue approximation, and 5-0 polybutester in a simple interrupted fashion to close the epidermis. Careful attention was paid to flap inset, tension, and contour to maintain nasal symmetry. Petrolatum and pressure dressing were applied postoperatively (Fig 3).

**Fig 4:**
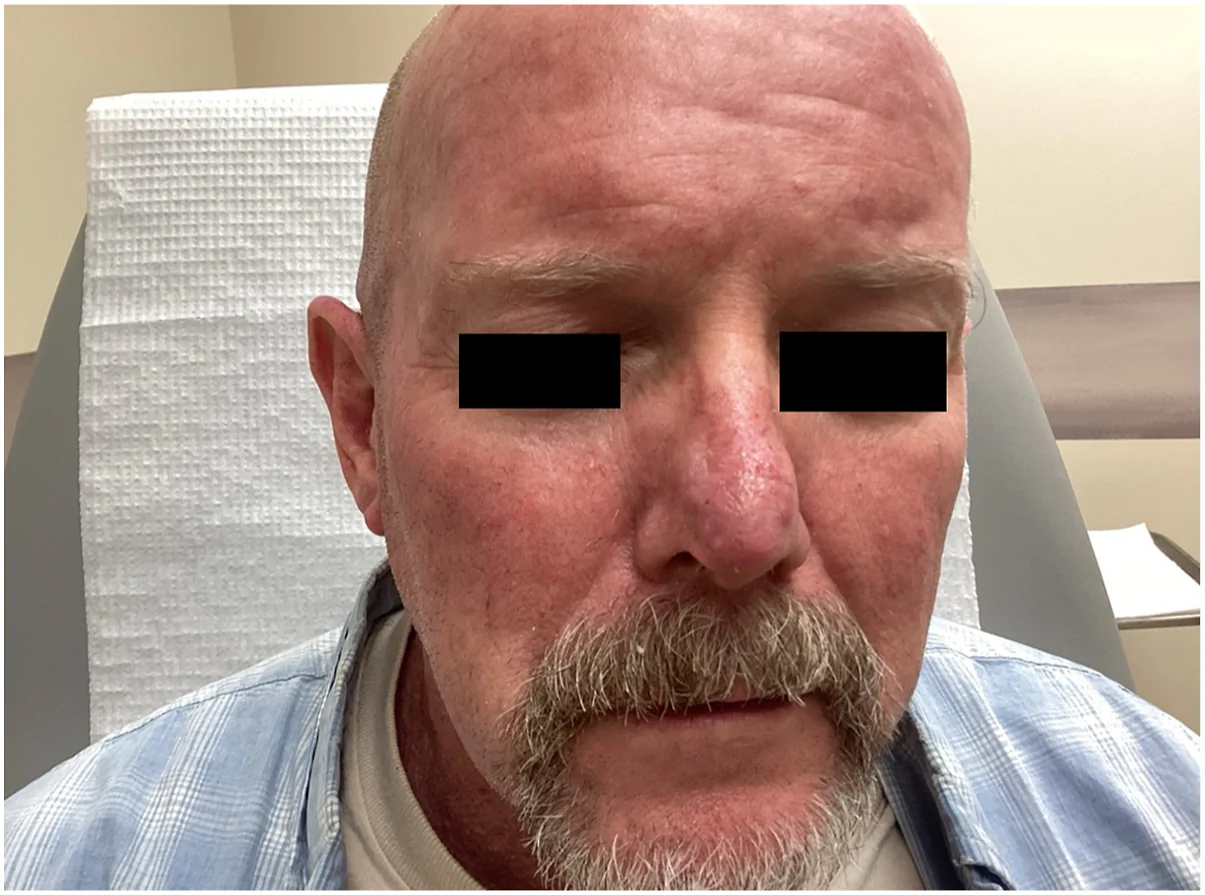
Six month follow-up evaluation of repair shows viable flap without evidence of necrosis or observable defect.

At the postoperative visit 4 weeks later, the patient had fully healed without complications. Follow-up examinations demonstrated resolution of the through-and-through defect, preservation of alar rim support, and satisfactory cosmetic and functional outcomes. At the 6-month postoperative visit, no flap necrosis, wound dehiscence, infection, or airway compromise was observed and there was no evidence of flap necrosis or return of through and through defect (Fig 4).

## Discussion

Full-thickness nasal infratip defects require thoughtful reconstruction to restore both form and function. Loss of internal nasal lining may predispose patients to alar collapse, notching, and airway obstruction if inadequately addressed.^6^ While cartilage grafting and staged procedures are often considered in such cases, these approaches may increase operative complexity and morbidity.^5^

Reconstruction of the distal nose remains uniquely challenging due to limited tissue laxity, relatively reduced vascular reserve, and high esthetic visibility.^4^ A recent systematic review and meta-analysis by Salzano et al evaluating surgical outcomes in distal nasal reconstruction reported overall complication rates ranging approximately 8% to 20% depending on reconstructive modality, with partial flap or graft necrosis representing one of the most common adverse events. Composite grafts and full-thickness skin grafts demonstrated higher rates of partial loss compared to well-vascularized local flaps, particularly in full-thickness defects where vascular support is limited.^7^ These findings underscore the importance of maximizing vascular reliability in distal nasal repairs, especially in revision settings.

Burow’s grafts remain a useful reconstructive option for select nasal defects; however, in full-thickness defects, they rely on plasmatic imbibition and inosculation without intrinsic blood supply, which may increase the risk of partial failure, especially in the distal nose where perfusion is comparatively reduced.^8^ These findings underscore the importance of maximizing vascular reliability in distal nasal repairs, particularly in revision settings, as illustrated in our case where partial graft failure necessitated secondary intervention. A hinge flap combined with a nasolabial transposition flap has previously been described for reconstruction of full thickness nasal alar defects, demonstrating the utility of vascularized local tissue for restoration of both the internal nasal lining and external skin.^9^ In contrast, our case describes a salvage reconstruction following partial Burow's graft failure. Rather than designing a separate hinge flap from adjacent tissue, the standing cone of the nasal tip rotation flap was intentionally preserved and repurposed to recreate the internal nasal lining. This modification allowed reconstruction using adjacent local tissue without requiring an additional donor site or cartilage graft.

This case highlights an alternative salvage technique utilizing tissue that is typically discarded during flap design. Repurposing the standing cone of a nasal tip rotation flap to reconstruct the internal nasal lining provided adequate structural support while avoiding additional donor sites or cartilage grafting. This approach was particularly advantageous in the setting of failed initial reconstruction, where preservation of remaining tissue and avoidance of further scarring were critical considerations.^10^

Burow’s grafts remain a useful reconstructive option for select nasal defects, although partial graft failure may occur in full-thickness defects due to limited vascular support.^2^ In such cases, secondary reconstruction using local flaps with modification of standard techniques can provide reliable outcomes.^4^ The technique described here offers a practical and reproducible option for dermatologic surgeons managing persistent through-and-through nasal infratip defects. One limitation of this technique is that it may not be suitable for larger full-thickness defects in which the defect size exceeds the available standing cone tissue needed to recreate the internal lining. Successful reconstruction also depends on preservation of a viable alar rim. If the alar rim is excessively thinned, structurally compromised, or absent, it may not adequately support or receive the rotation flap. In these settings, alternative reconstructive approaches with cartilage grafting or staged repair may be necessary.^6^

### Declaration of generative AI and AI-assisted technologies in the writing process

No artificial intelligence tools were used in the writing, analysis, or preparation of this case report.

## Conflicts of interest

None disclosed.
